# Data on amyloid precursor protein accumulation, spontaneous physical activity, and motor learning after traumatic brain injury in the triple-transgenic mouse model of Alzheimer׳s disease

**DOI:** 10.1016/j.dib.2016.08.041

**Published:** 2016-08-26

**Authors:** Yasushi Kishimoto, Hajime Shishido, Mayumi Sawanishi, Yasunori Toyota, Masaki Ueno, Takashi Kubota, Yutaka Kirino, Takashi Tamiya, Nobuyuki Kawai

**Affiliations:** a Laboratory of Neurobiophysics, Kagawa School of Pharmaceutical Sciences, Tokushima Bunri University, Japan; b Department of Neurological Surgery, Faculty of Medicine, Kagawa University, Japan; c Department of Inflammation Pathology, Faculty of Medicine, Kagawa University, Japan; d Department of Neurological Surgery Kagawa General Rehabilitation Hospital, Japan

**Keywords:** AD, Alzheimer׳s disease, ANOVA, analysis of variance, APP, amyloid precursor protein, CR, conditioned response, CS, conditioned stimulus, TBI, traumatic brain injury, US, unconditioned stimulus, Traumatic brain injury, Eyeblink conditioning, Amyloid precursor protein, Spontaneous physical activity, Rotarod test

## Abstract

This data article contains supporting information regarding the research article entitled “Traumatic brain injury accelerates amyloid-β deposition and impairs spatial learning in the triple-transgenic mouse model of Alzheimer׳s disease” (H. Shishido, Y. Kishimoto, N. Kawai, Y. Toyota, M. Ueno, T. Kubota, Y. Kirino, T. Tamiya, 2016) [1]. Triple-transgenic (3×Tg)-Alzheimer׳s disease (AD) model mice exhibited significantly poorer spatial learning than sham-treated 3×Tg-AD mice 28 days after traumatic brain injury (TBI). Correspondingly, amyloid-β (Aβ) deposition within the hippocampus was significantly greater in 3×Tg-AD mice 28 days after TBI. However, data regarding the short-term and long-term influences of TBI on amyloid precursor protein (APP) accumulation in AD model mice remain limited. Furthermore, there is little data showing whether physical activity and motor learning are affected by TBI in AD model mice. Here, we provide immunocytochemistry data confirming that TBI induces significant increases in APP accumulation in 3×Tg-AD mice at both 7 days and 28 days after TBI. Furthermore, 3×Tg-AD model mice exhibit a reduced ability to acquire conditioned responses (CRs) during delay eyeblink conditioning compared to sham-treated 3×Tg-AD model mice 28 days after TBI. However, physical activity and motor performance are not significantly changed in TBI-treated 3×Tg-AD model mice.

**Specifications Table**

**Table:** 

Subject area	*Neuroscience*
More specific subject area	*Alzheimer*׳*s disease, immunohistochemistry, behavioral neuroscience*
Type of data	*Graph, figure, image*
How data was acquired	*Behavioral phenotyping (HomeCageScan, CleverSys Inc., Reston, VA; rotarod system (Ugo Basile, Monvalle VA, Italy); eyeblink conditioning system (A-M Systems, Sequim, WA, USA) and fluorescent microscope (ECLIPS Ci, Nikon, Tokyo, Japan).*
Data format	*Raw* and *analyzed*
Experimental factors	*5- to 7-month-old homozygous triple-transgenic AD-model (3*×*Tg-AD) mice*
Experimental features	*3*×*Tg-AD mouse were examined using behavioral and immunohistochemical methods following TBI or sham treatment*
Data source location	*Miki-cho, Kagawa, Japan and Sanuki-shi, Kagawa, Japan*
Data accessibility	*Data is within this article*

**Value of the data**

• These data describe specific behavioral and histological changes that occur in an Alzheimer׳s disease (AD) mouse model after traumatic brain injury (TBI), which may serve as a reference for other researchers interested in examining neurodegeneration after TBI in AD.
• These data serve as a fundamental and useful resource for other researchers that may seek to describe the progression of AD after brain injury; these data may stimulate further study of the mechanism of neurodegeneration that occurs after TBI in AD.
• This dataset also offers basic information regarding the use of classical eyeblink conditioning after TBI in an AD mouse model, which may be useful to other researchers in the development of future experiments.

## 1 Data

**Fig. 1:**
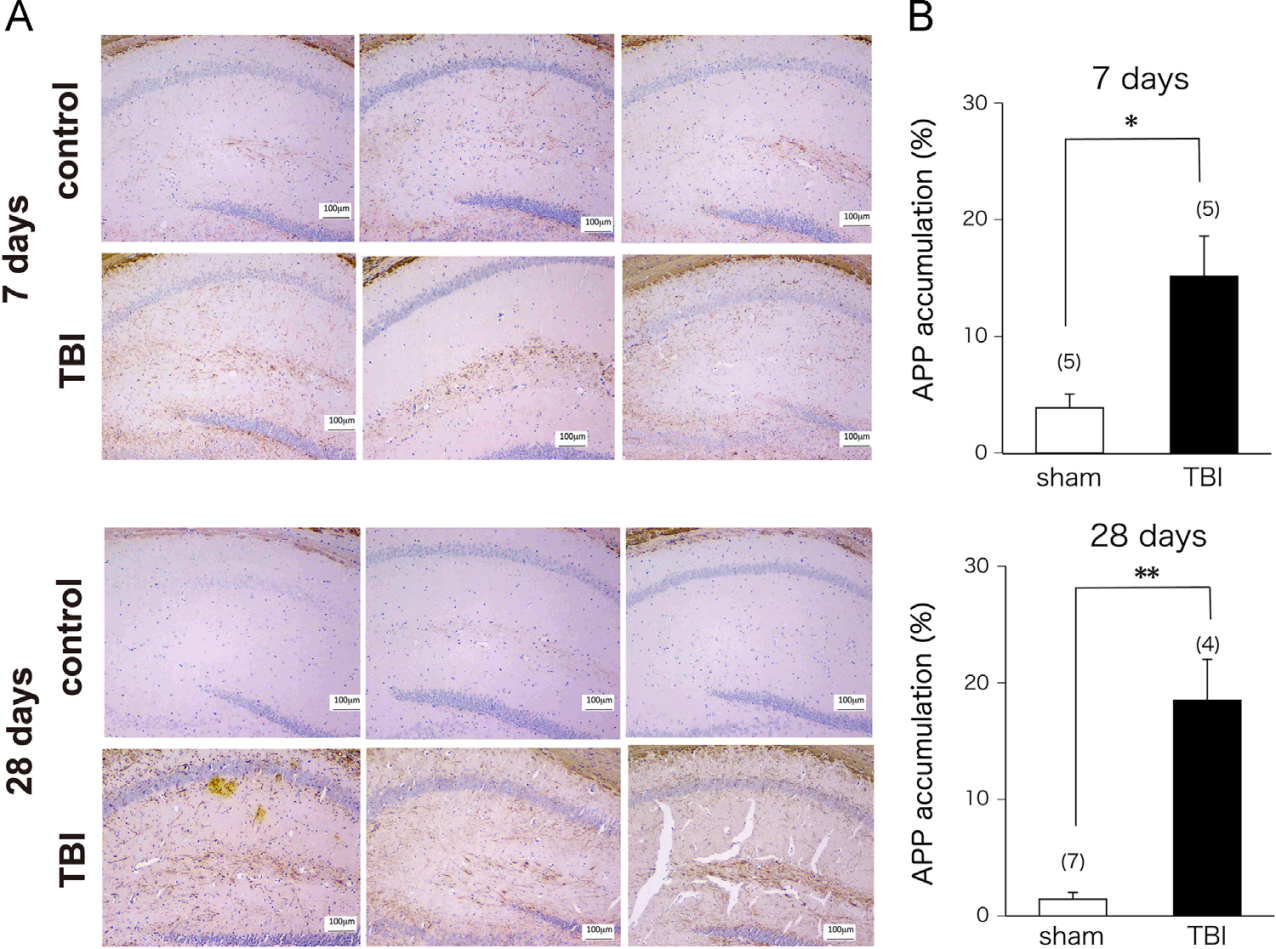
Data showing amyloid precursor protein (APP) accumulation in the 3×Tg-Alzheimer׳s disease (AD) model mouse hippocampus after traumatic brain injury (TBI). (A) Images demonstrate axonal immunoreactivity for APP in the hippocampal commissure at 7 days or 28 days after TBI. Scale bars: 100 μm. (B) Quantification of hippocampal APP accumulation. The presence of APP (expressed as the percentage of the area occupied by APP-immunopositive deposition in the ipsilateral hippocampus) was assessed using the ImageJ analysis system (National Institute of Health, Bethesda, MD, USA). APP accumulation was significantly greater in TBI-treated 3×Tg-AD (closed bar) mouse hippocampus both 7 days and 28 days after injury. **p<0.01, *p<0.05 relative to the corresponding sham-operated control group (open bar).

**Fig. 2:**
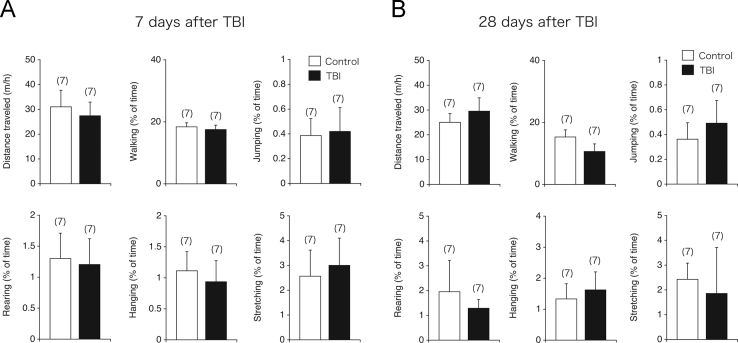
Data showing spontaneous physical activity (SPA) measures in traumatic brain injury (TBI)-treated 3×Tg-Alzheimer׳s disease (AD) model mice. (A, B) SPA was evaluated in TBI-treated (closed bar) and sham-treated 3×Tg-AD model (open bar) mice at 7 days (A) or 28 days (B) after TBI (*n*=7 in each group). Six separate parameters of spontaneous behavior (distance traveled, walking, jumping, rearing, hanging, and stretching) were evaluated for 3 h in the home cage. There were no significance differences between sham-operated and TBI-treated 3×Tg-AD model mice on any of the behavioral measures.

**Fig. 3:**
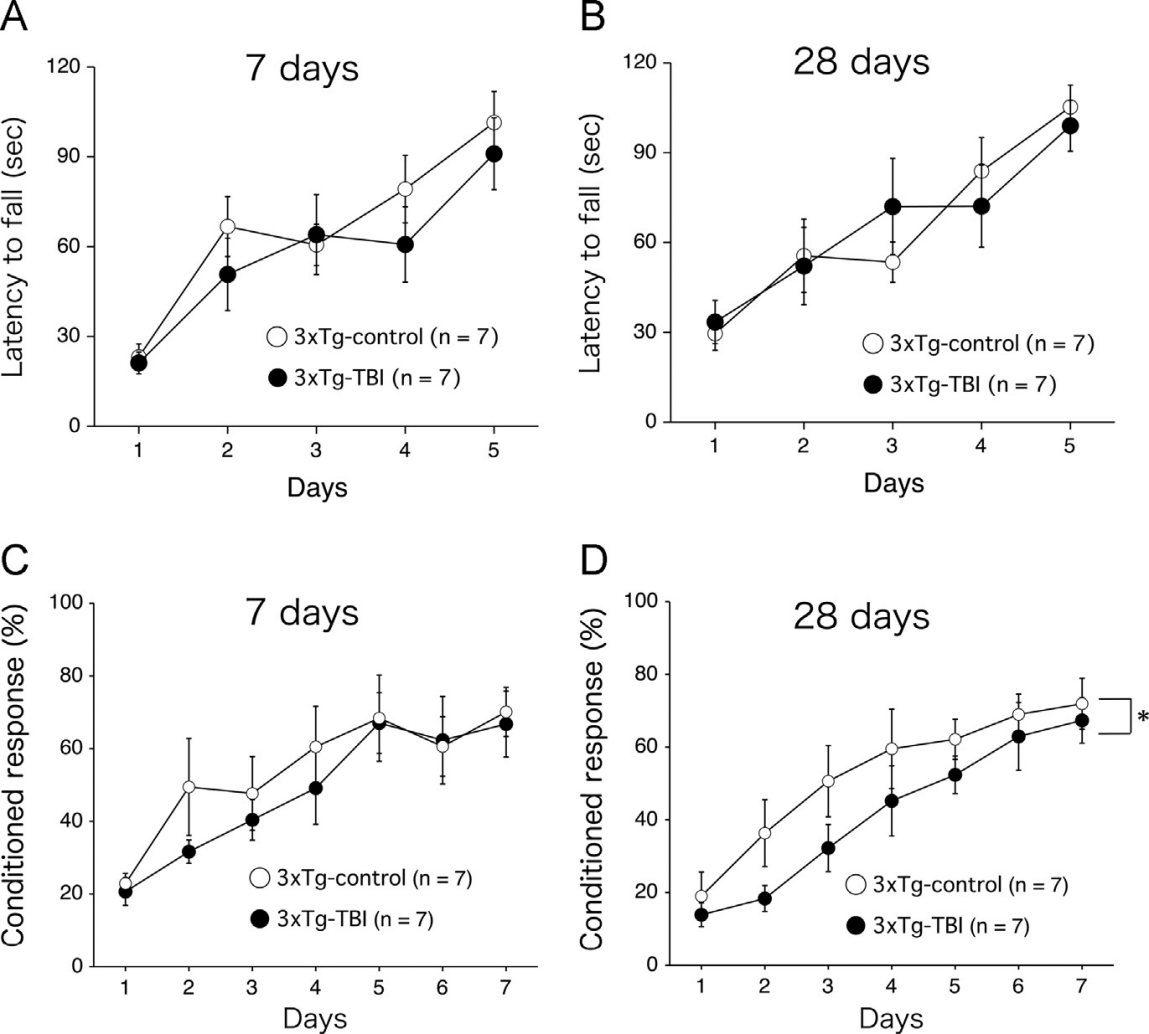
Data showing motor coordination and motor learning after traumatic brain injury (TBI) as assessed by the rotarod and delay eyeblink conditioning tasks, respectively. (A, B) 3×Tg-Alzheimer׳s disease (AD) mice (*n*=14) were equally assigned to the sham-operated or TBI-treated groups (*n*=7 per group), and then were subjected to the rotarod test. (A) There was no significant difference in rotarod performance between sham-operated 3×Tg-AD mice and TBI-treated mice at 7 days after injury. Analysis of variance (ANOVA) revealed no significant interaction effects between sessions and groups [*p*=0.68; *F*(4, 48)=0.571] and no significant group effect [*p*=0.371; *F*(1, 12)=0.870]. (B) There was also no significant difference in rotarod performance between sham-operated 3×Tg-AD mice and TBI-treated mice at 28 days after injury. ANOVA revealed no significant interaction effects between sessions and groups [*p*=0.61; *F*(4, 48)=0.683] and no significant group effect [*p*=0.98; *F*(1, 12)=0.00058]. (C, D) 3×Tg-AD mice (*n*=14) were equally assigned to sham-operated or TBI-treated groups (*n*=7 per group), and then were subjected to the delay eyeblink conditioning task. (C) There were no significant differences in the ability to acquire the conditioned response (CR) during the 7-day sessions between the 2 groups at 7 days after TBI. ANOVA revealed no significant interaction effects between sessions and groups [*p*=0.29; *F*(6, 72)=0.436] and no significant group effect [*p*=0.492; *F*(1, 12)=0.500]. (D) In contrast, there were significant differences in the ability to acquire the CR between the 2 groups at 28 days after TBI. ANOVA revealed no significant interaction effects between sessions and groups [*p*=0.91; *F*(6, 72)=0.336], but a significant group effect was observed [*p*=0.023; *F*(1, 12)=6.755]. However, a post hoc Bonferroni test revealed no significant differences between the two groups on any day. *p<0.05 relative to the corresponding sham-operated control group.

We first examined whether traumatic brain injury (TBI) induces amyloid precursor protein (APP) accumulation in the triple-transgenic AD-model (3×Tg-AD) mouse hippocampus (Fig. 1). Next, we assessed the short-term and long-term effects of TBI on spontaneous physical activity, motor coordination, and motor learning in 3×Tg-AD mice (Fig. 2, Fig. 3).

## 2 Experimental design, materials and methods

### 2.1 Animals

Behavioral and histological data were obtained from 5- to 7-month-old 3×Tg-AD mice (25–30 g) [2].

### 2.2 Traumatic brain injury

3×Tg-AD mice were subjected to a sham operation or TBI using a weight-drop method [1]. We evaluated APP accumulation in the hippocampus of these mice at either 7 or 28 days after injury. A second group of 3×Tg-AD mice was also subjected to a sham operation or TBI, but behavioral measures were alternately collected at either 7 days or 28 days after injury.

### 2.3 Antibody staining

APP accumulation was examined using immunostaining with anti-APP polyclonal antibody (1:1000; AnaSpec, Fermont, CA). Briefly, sections were subjected to an antigen retrieval step by immersing the sections in 90% formic acid for 10 min before immunohistochemistry for Aβ. Sections were washed in diluted water, and endogenous peroxidases were quenched using a freshly prepared mixture of methanol (150 ml) plus hydrogen peroxide (33%, 1.5 ml) for 30 min and 2% normal goat serum in phosphate-buffered saline (PBS) with 0.1% Tween-20 for 30 min. Sections were then incubated overnight with a polyclonal antibody to APP at 37 °C. Sections then were rinsed five times for 5 min each with PBS with 0.1% Tween-20, and then incubated with peroxidase-labeled anti-rabbit antibody (Histofine Simple Stain Max PO, Nichirei, Japan) for 30 min at 37 °C. Peroxidase activity was detected with diaminobenzidine (DAB, Nichirei, Japan) for visualization. Sections were counterstained with hematoxylin and dehydrated. Negative controls included the application of the same immunohistochemistry protocol to sections, except PBS was applied instead of the primary antibody.

### 2.4 Image analysis of APP positive cells

The method employed here was essentially the same as that described previously [1]. Each image was analyzed using image analysis software (ImageJ 1.50i, National Institutes of Health, Bethesda, MD, USA). Areas occupied by APP-immunoreactive products within the regions of interest were evaluated; the total area occupied by the outlined structures was measured in order to calculate the percentage of the area occupied by the immunoreactive products over the total outlined anatomical area in the image.

### 2.5 Behavioral phenotyping

All behavioral studies were performed blind to genotype. All behavioral experiments were conducted during the light phase. All behavioral tests were initiated at either 7 days or 28 days after TBI.

#### 2.5.1 Spontaneous physical activity

The method employed here was essentially the same as that described previously [3], [4]. Mice were transferred to a familiar home cage (21×31×12 cm) and were video recorded for 3 h, beginning from 10:00 to 14:00. Movie data were analyzed using the CleverSys HomeCageScan system (CleverSys Inc., Reston, VA, USA), and spontaneous physical activities such as distance traveled, rearing, hanging, and stretching were evaluated.

#### 2.5.2 Rotarod test

The rotarod apparatus (Ugo Basile, Monvalle VA, Italy) consisted of a rod 3 cm in diameter, rotating at 20 rpm. The time that the mouse remained on the rod was measured (a maximum of 120 s). Mice completed one session every day for 5 days (2 trials per session).

#### 2.5.3 Eyeblink conditioning

Eyeblink conditioning procedures were essentially the same as previously described [3]. Under anesthetized conditions, four stainless steel wires (100 μm in diameter; A-M Systems, Sequim, WA, USA) were subcutaneously implanted under the left eyelid. Two wires were used to apply the unconditioned stimulus (US), and the remaining two wires were used to receive an electromyogram (EMG) signal from the musculus orbicularis oculi. A 352-ms tone (1 kHz, 80 dB) generated by a speaker was used as the conditioned stimulus (CS), and a 100-ms electrical shock (0.2–0.5 mA, 100-Hz) was used as the US. Each daily session consisted of 100 trials (10 trials of the CS alone and 90 trials of the CS and US paired together). In the present study, the CS and the US temporally overlapped and terminated simultaneously. Mice completed 7 days of these acquisition sessions. The EMG signals were analyzed the same way as previously described [3], [5], [6].

## 3 Statistical analysis

Statistical analyses were conducted using the Statistical Package for GraphPad Prism 6 (GraphPad Software Inc., La Jolla, CA, USA) to determine differences between groups. Specifically, two-way repeated measures analysis of variance (ANOVA) was used to analyze the data, followed by a post hoc Bonferroni test and unpaired *t*-tests (two-tailed). Significance was defined as *p*<0.05.
